# Remarkable Case of Frequent Fractures of Femurs with the Treatment of the Eighth Fracture

**Published:** 1884-04

**Authors:** D. H. Howell

**Affiliations:** Atlanta, Ga.


### A REMARKABLE CASE OF FREQUENT FRACTURES OF FEMURS, WITH THE TREATMENT OF THE EIGHTH FRACTURE.

BY D. H. HOWELL, M. D., ATLANTA, GA.

The object of this paper is simply the report of a case in which I have felt great interest, and which, from its rarity, will, I feel, be of interest to the profession.

On the night of the 30th of November, 1883, I was called to see Mr. M. A. B., a young white man of this city, who had sustained a fracture of his left femur, middle third. The history that the young man gives is quite remarkable. He is now twenty-eight years old, and his general health is excellent and has always been. He is only four feet five inches in height, and weighs about eighty pounds. I can discover no syphilitic taint, and no diathesis with any signs other than the predisposition to fractures of his femurs. Whether all of his bones have this characteristic I cannot say, but no other than the femurs have shown signs of extraordinary brittleness. These misfortunes began as early as his second year, the first one, which was of the left thigh, being caused by falling from a chair. His physician supposed this fracture to be of the surgical neck. A few years later, while playing with a dog, he was tripped, and the simple fall upon the ground broke his right thigh in the same region as the previous fracture. One year later, a simple fall produced a second fracture of the left femur, in the region of the first. Two years later, while walking a plank six inches from the ground, he fell and sustained a second fracture of the right thigh in the same region as the first fracture of that bone. At the age of twelve, he was pushed down four steps by a school-mate, and his right thigh was broken for the third time, in the same region. A year or two later, he was thrown down upon the smooth ground by his brother, and his left thigh was broken for the third time in the region of the previous fractures. This made three fractures for each femur. The seventh fracture was of the right femur, and occurred in his 16th year. He and several of his school-mates were standing upon a rock wall about four feet high. They were seeing who could jump the farthest, his turn came last, he jumped, and his right femur was fractured for the fourth time. The eighth and last, and the one I treated, occurred in his twenty-eighth year, and was caused by his falling over the rail which is placed across the end of a side-track to prevent engines and cars from running off the end of the track. This was a severe fall, and resulted in the fourth fracture of left femur in the middle third.

These fractures all must have been well treated, for no deformity other than a slight natural curvature outwards, is discernible, at least I suppose the curvature is natural, for there is a uniformity about it which one would hardly expect the results of badly treated fractures to produce. In the treatment of the eighth fracture he was made comfortable by placing his leg on a double inclined plane made of pillows, where it remained for 48 hours. At the expiration of that time the plaster of paris splint was applied, from the middle of the leg to as near the perineum as comfort would allow. I let this remain for three weeks, and on removing it, found that no union had taken place. My fears that it would never unite, which were strong at first, grew stronger. Another splint was applied immediately, which, from a fault in the plaster of paris, was so heavy that it was impossible for him to move with any degree of ease. I allowed this to remain only for one week when another was applied of the desired thickness and weight. This allowed him to move with comfort, and it remained in position for forty days, when it was removed and the bone found to be united. Before the removal of the last splint, he could move about the house by the aid of his crutches, and could partly rest himself on his fractured thigh. This he was able to attempt before the bone had thoroughly united, as the exact fit and the immovable nature of the splint would allow. It is in fact, my opinion that the bone would never have united if he had been treated by some method which would have prevented, at least a moderate exercise of his body, and especially his fractured limb. He is at present able to attend his usual business, and his limb appears to be well united, and as strong as before the fracture.

